# Small non-coding RNA profiling in human biofluids and surrogate tissues from healthy individuals: description of the diverse and most represented species

**DOI:** 10.18632/oncotarget.23203

**Published:** 2017-12-14

**Authors:** Giulio Ferrero, Francesca Cordero, Sonia Tarallo, Maddalena Arigoni, Federica Riccardo, Gaetano Gallo, Guglielmo Ronco, Marco Allasia, Neha Kulkarni, Giuseppe Matullo, Paolo Vineis, Raffaele A. Calogero, Barbara Pardini, Alessio Naccarati

**Affiliations:** ^1^ Department of Computer Science, University of Turin, Turin, Italy; ^2^ Department of Clinical and Biological Sciences, University of Turin, Turin, Italy; ^3^ Italian Institute for Genomic Medicine, IIGM (formerly Human Genetics Foundation, HuGeF), Turin, Italy; ^4^ Molecular Biotechnology Center, Department of Biotechnology and Health Sciences, University of Turin, Turin, Italy; ^5^ Department of Medical and Surgical Sciences, University of Catanzaro, Catanzaro, Italy; ^6^ Department of Colorectal Surgery, Clinica S. Rita, Vercelli, Italy; ^7^ Center for Cancer Epidemiology and Prevention, AO City of Health and Science, Turin, Italy; ^8^ Department of Surgical Sciences, University of Turin and Città della Salute e della Scienza, Turin, Italy; ^9^ Department of Medical Sciences, University of Turin, Turin, Italy; ^10^ MRC-HPA Centre for Environment and Health, School of Public Health, Imperial College London, London, United Kingdom; ^11^ Department of Molecular Biology of Cancer, Institute of Experimental Medicine, Prague, Czech Republic

**Keywords:** next-generation sequencing, small non-coding RNA profiling, microRNAs, non-invasive biomarkers, surrogate tissues

## Abstract

The role of non-coding RNAs in different biological processes and diseases is continuously expanding. Next-generation sequencing together with the parallel improvement of bioinformatics analyses allows the accurate detection and quantification of an increasing number of RNA species. With the aim of exploring new potential biomarkers for disease classification, a clear overview of the expression levels of common/unique small RNA species among different biospecimens is necessary. However, except for miRNAs in plasma, there are no substantial indications about the pattern of expression of various small RNAs in multiple specimens among healthy humans.

By analysing small RNA-sequencing data from 243 samples, we have identified and compared the most abundantly and uniformly expressed miRNAs and non-miRNA species of comparable size with the library preparation in four different specimens (plasma exosomes, stool, urine, and cervical scrapes).

Eleven miRNAs were commonly detected among all different specimens while 231 miRNAs were globally unique across them. Classification analysis using these miRNAs provided an accuracy of 99.6% to recognize the sample types. piRNAs and tRNAs were the most represented non-miRNA small RNAs detected in all specimen types that were analysed, particularly in urine samples. With the present data, the most uniformly expressed small RNAs in each sample type were also identified. A signature of small RNAs for each specimen could represent a reference gene set in validation studies by RT-qPCR.

Overall, the data reported hereby provide an insight of the constitution of the human miRNome and of other small non-coding RNAs in various specimens of healthy individuals.

## INTRODUCTION

The discovery of many stable extracellular small RNAs has changed our view of gene expression regulation, including the role that these molecules may play in several complex processes previously partially understood such as cell-to-cell communication [1]. In this respect, with an astonishing number of publications in the last decade, microRNAs (miRNAs) represent the most explored small non-coding RNA (sncRNA) species in humans [2]. A large number of studies has demonstrated that cellular and extracellular miRNA altered expression is associated with a wide variety of diseases, including cancer [3, 4]. However, little is known about the presence within the same matrix of other common species of sncRNAs such as piwi-interacting RNAs (piRNAs), small nucleolar RNAs (snoRNAs), tRNAs etc. All these versatile RNA species are known to be key components of molecular interactions and gene regulation in eukaryotes [5].

The field of circulating extracellular RNA molecules is rapidly growing thanks to the implementation of Next-Generation Sequencing (NGS) technologies and bioinformatics solutions that analyze the huge amount of data released from sequencing. With such high-throughput approach, all extracellular RNAs can be quantified and tested as potential sources of new diagnostic and therapeutic biomarkers in many different types of biological samples [6]. To achieve this, RNA-Sequencing (RNA-Seq) has emerged as a powerful tool in transcriptomics, gene expression profiling and biomarker discovery. Sequencing cell-free nucleic acids from liquid biopsies additionally provides exciting possibilities for molecular diagnostics, and might help establish disease-specific biomarker signatures [7]. Lower complexity, not known post-processing modifications, simple detection and amplification methods, tissue-restricted expression profiles, and sequence conservation between humans and model organisms make extracellular miRNAs and other sncRNAs ideal candidates for non-invasive biomarkers to reflect and study various physiopathological conditions in the body [8]. It is possible to extract and quantify high-quality sncRNAs from a wide range of cell and tissue sources, including cell lines, fresh and formalin-fixed paraffin-embedded tissues, plasma, serum, urine and other body fluids [8–10]. Despite this increasing interest, the field is still largely in an exploratory and descriptive phase. There are no standardized methods for sample collection, isolation, or analysis. There is also no general agreement on the terms for a good quality sample definition, and each specimen (body fluid or surrogate tissue) under various disease/injury conditions are likely to have diverse contents and different criteria for quality assessment [7, 11]. A growing number of isolation methods for profiling circulating extracellular RNA molecules have been developed but still, there is no gold standard for the most efficient inclusive or selective protocols [6]. However, the complexity of the small RNA-Seq workflow bears challenges and biases that researchers need to be aware of, in order to generate high-quality data [12].

The creation of large repositories including data from different human specimens, isolation methods, detection platforms, and analysis tools is essential to increase our understanding of the extent and types of extracellular RNA material present in different body fluids/surrogate tissues. At present, there are few large datasets describing the extracellular contents in biofluid samples from healthy controls [13–17]. Besides, previous studies on extracellular sncRNAs have investigated very small numbers or pooled samples with the purpose of identifying a specific class of RNAs [18]. The largest investigations of samples focused almost exclusively on miRNAs, with the main limitation of measuring either only targeted miRNAs in large numbers of individuals or the whole known miRNome in very small populations. In a recent work it has been described the largest group of plasma-based miRNAs and the first broadest variety of extracellular (non-miRNA) sncRNAs in a large population [15]. In another similar work, authors profiled the small RNA (16–32 nts) payload of human biofluids by NGS. Extracellular RNAs were isolated from plasma, urine and saliva samples from 55 young male athletes and sequenced to establish a sncRNA pattern at steady state [6].

In the present study, we investigated pattern and expression levels of miRNAs and other sncRNAs of comparable size in four different biospecimens representing ideal surrogate tissues for diagnostic and screening programs. Specifically, we analysed data from small RNA-Seq from 125 plasma-derived exosomes, 48 urine, 31 cervical scrapes, and 39 stool samples collected from healthy subjects. For cervical scrapes and stool, this is the first study investigating sncRNAs by NGS. In addition, urine and stool samples were paired with those from plasma collected from the same subjects.

## RESULTS

### Overview of study samples and pipeline analysis

We analysed small RNA-Seq data of RNA extracted from exosomes from 125 plasma samples of healthy donors derived from three different studies (respectively 39 for the Study 1, 46 for the Study 2, and 40 for the Study 3) (Materials and Methods). Additionally, sequencing was performed on RNA from 39 faecal samples (Study 1), 48 urine samples (Study 2), and cervical scrapes from 31 Human Papilloma Virus (HPV) negative women. Some of the plasma sample donors provided at the same occasion a sample of stool (39 from Study 1) or urine (46 from Study 2).

Total RNA was isolated from samples with specific kits for each type of specimens while library preparation for small RNA-Seq was performed adopting the same kit and protocol. Libraries were run at the same sequencing facility. Finally, all bioinformatics analyses (i.e. pre-processing of raw data) were performed following the same pipeline by the same operator.

**Figure 1:**
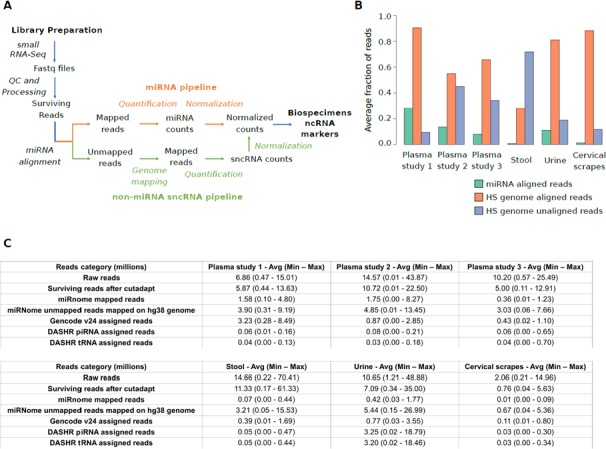
(**A**) Schematic representation of the computational pipeline applied in the analysis of small RNA-Seq dataset from healthy individuals. The modules of the pipeline designed for miRNAs and other sncRNAs are depicted in orange and green, respectively. (**B**) Bar plot showing for each specimen, the average number of sequencing reads aligned to miRNA annotations (green), unmapped on miRNA annotations but mapped on human genome (red), and unmapped on both miRNA annotations and the human genome (blue). (**C**) Table reporting the average, minimum, and maximum number of reads (in million) composing the starting datasets, aligned in the different analysis phases, or assigned to specific RNA annotations. HS= Homo sapiens.

To explore the landscape of sncRNA expression levels in different biospecimens, we designed a computational strategy for small RNA-Seq data analysis (Figure 1A). We updated the miRNA analysis pipeline published by our group [19] by adding a second phase focused on the analysis of small RNA-Seq reads unmapped against the human miRNome (Materials and Methods).

Initially, small RNA-Seq datasets were pre-processed and quality controlled to remove adapter sequences and low-quality reads. The processing information about the 243 datasets analysed is provided in Supplementary Table 1A and 1B. Quality check confirmed that were no reads shorter than 15 nucleotides and the rate of low quality reads (quality score < 30) was on average below 8%, with urine and stool samples providing the best rates (<1%).

### Identification of miRNAs and non-miRNA sncRNAs

miRNA mapping analysis showed remarkable differences among specimens for read alignment rates (Figure 1B, 1C and Supplementary Figure 1A). Consistently with the highest rates of read alignment (Figure 1C), urine samples were generally associated with a high number of reads (median reads = 12.38 million) followed by plasma exosomes (median reads = 11.34 million), stool (median reads = 4.88 million), and cervical scrapes (median reads = 4.13 million) (Supplementary Figure 2A).

Datasets from plasma exosome and urine samples were characterized by the highest miRNA alignment rates (16.3% and 11.0%, of reads aligned, respectively) while datasets from stool and cervical scrape samples were associated on average with low miRNome alignment rates (0.7% and 1.2% of reads aligned, respectively).

Of the 1,823 miRNA annotations from miRBase, a range from 19.9% (cervical scrapes) to 73.8% (plasma exosomes *study* 2) of human miRNAs were detected in all the investigated specimens. A median of 58.61% of miRBase annotations were detected across the four specimen types. Specifically, miR-486-5p was the most expressed miRNA in plasma exosomes samples (median reads = 180,173 reads) while miR-320a (median reads = 198 reads), miR-6813-5p (median reads = 5,911 reads), and miR-30a-5p (median reads = 25,910 reads) were the highest expressed in cervical scrapes, stool, and urine, respectively (Supplementary Figure 2B).

Since a large fraction of sequencing reads did not map on miRNome (Supplementary Table 1A), the alignment analysis was extended to other candidate sncRNA annotations by initially remapping reads on the human genome. Then, mapped reads were assigned to sncRNA annotations quantifiable using our size selection criterion. These annotations included sncRNAs annotated in GENCODE v24 database [20] (transcript length ≤70 bp) as well as piRNA (average length 31±1 bp) and tRNA (average length 74±7 bp) species annotated in the Database of Small Human non-coding RNAs (DASHR) release 1 [21] (Supplementary Table 1C). The alignment rates observed were higher for cervical scrapes (88.4%) followed by urine (81.1%), and plasma exosome samples (69.5%). As expected, stool datasets were associated with the lowest alignment rate on the human genome (28.1%) consistently with the presence of microbiome RNAs and other RNAs introduced by the diet, contributing to the large fraction of faecal RNA content (Supplementary Table 1A and Supplementary Figure 1A). In urine, most reads were assigned to piRNA (44.5%) or tRNA annotations (45.1%). Conversely, in the other specimens, a low assignment rate was observed ranging 1.8–3.4% for piRNAs and 1.0–3.3% for tRNAs, respectively (Supplementary Figure 1B). Homologous piRNAs annotated to different loci were associated with the same number of reads across samples.

### Common and specific miRNAs among different specimens

**Figure 2:**
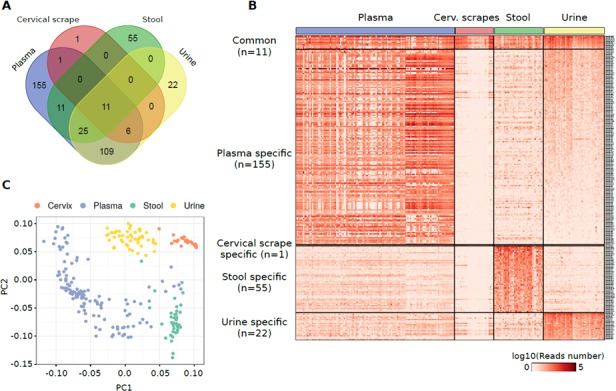
(**A**) Venn diagram reporting the number of miRNAs detected in different specimens from healthy individuals and their overlap. (**B**) Heat map showing the log10 number of normalized reads supporting the miRNAs specifically detected in one specimen or commonly detected among them. (**C**) PCA plot showing the small RNA-Seq datasets separation obtained using miRNAs detected in samples analysed.

Considering the individual datasets from plasma exosome samples, it was evident a study-specific influence on the read alignment distribution with samples from the *study 1* characterized, on average, by the overall highest alignment on miRNome annotations (28.3% aligned reads). However, PCA on miRNAs and other sncRNA annotations expressed in at least one study (within study median number of reads >20) showed a distinct cluster formed by all plasma exosome samples with respect to other biospecimens (Supplementary Figure 1C). A comparable result was obtained by computing a pairwise correlation analysis: datasets from the three plasma exosome studies clustered together and were clearly separated from the others (Supplementary Figure 1D). Given the results from the PCA and correlation analyses, plasma exosome samples from the three studies were merged into a single group after read count correction with Surrogate Variable Analysis (SVA). The identification of pattern of miRNAs detectable in the different specimens was performed by considering miRNAs characterized by a median of normalized reads higher than 20 in at least one specimen. Using this threshold, cumulatively, 394 miRNAs were quantified in at least one specimen (Figure 2A, Supplementary Table 2A). Eleven miRNAs were identified as commonly detectable in all types of specimens: miR-320a, miR-589-5p, miR-636, miR-1273a, miR-3960, miR-4419a, miR-4497, miR-4709-5p, miR-4792, miR-7641-1, and miR-7641-2.

Functional enrichment analysis of validated target genes of the 11 shared miRNAs revealed biological processes related to mRNA translation and transcription including translational initiation (GO:0006413, *p* = 1.9 × 10^–8^) or positive regulation of transcription, DNA-templated (GO:0045893, *p* = 4.2 × 10^–7^) (Supplementary Table 2B).

Plasma exosome samples were characterized by the highest number of specimens-specific miRNAs (155 miRNAs) followed by stool (55 miRNAs), urine (22 miRNAs), and cervical scrape samples (one miRNA) (Figure 2A, 2B). Considering only the specimen-specific miRNAs, miR-122-5p was the most expressed in plasma exosome samples (median reads = 32,512 reads) while miR-655-5p (median reads = 792 reads), miR-204-5p (median reads = 750 reads) and miR-4741 (median reads = 28 reads) were the most abundantly expressed in stool, urine, and cervical scrapes, respectively (Supplementary Figure 2C).

PCA analysis of the highly-expressed sets of miRNAs showed a good accuracy in the classification of different biospecimens (Figure 2C). To identify the discriminative miRNAs in the specimen classification, we also performed a classification and attribute selection analysis. Using a Random Forest classifier, we obtained an accuracy of 99.6% with only one sample incorrectly classified (Supplementary Table 2C). All the miRNAs analysed were associated with a high chi-square statistic (merit) in the attribute selection analysis with miR-204-5p, miR-5698, and miR-335-3p associated with the highest merit (Supplementary Table 2D).

For a subset of patients, paired data from plasma and stool samples or from plasma and urine samples were available allowing a comparison between expression levels of sncRNAs in the different specimens from the same subject. As reported in Supplementary Table 2E, 2F, a low co-expression was generally observed either between plasma-stool or plasma-urine samples. The only exception was miR-3665 which was characterized by a positive correlation between plasma and urine samples (*r =* 0.59, *p* = 2.0 × 10^–5^).

Prediction of candidate miRNA isoforms (isomiRs) was also performed using our datasets. As reported in Supplementary Table 2G, 832 isomiRs associated with more than 20 supporting reads in at least one specimen type were detected. Overall, 94.4% of isomiRs were detected in plasma exosome or urine samples consistently with the higher number of aligned reads in these samples. The isomiRs with the highest number of supporting reads were a 3′ variant of miR-486/miR-486-2 in plasma samples, a 5′ variant of miR-934 in urine sample, a 5′ variant of miR-7704 in cervical scrapes, and a 3′ variant of miR-583 in stool samples. Among the previously identified 11 common miRNAs, eight were associated to an isomiR predicted in only one or two types of specimens (particularly in plasma or urine samples) (Supplementary Table 2H).

### Expression pattern of other sncRNAs

**Figure 3:**
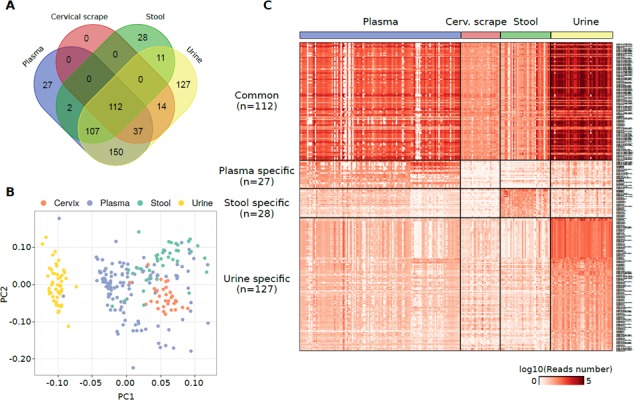
(**A**) Venn diagram reporting the number of non-miRNA sncRNA species detected in different specimens from healthy individuals and their overlap. (**B**) PCA plot showing the small RNA-Seq datasets separation obtained using the non-miRNA RNA species detected in the samples analysed. (**C**) Heat map showing the log10 number of normalized reads supporting the non-miRNA RNA species detected in one specimen only or commonly detected among them.

Cumulatively, 615 non-miRNA sncRNAs were quantified in at least one specimen. Of this set of annotations, 112 sncRNAs were commonly detected in all the analysed sample types (Figure 3A and Supplementary Table 3A). Coherently with the highest alignment rates, piRNAs were the most represented type of sncRNAs in urine, plasma exosomes, and stool (Supplementary Figure 2D). Urine samples emerged as the specimen characterized by the highest piRNA and tRNA contents (Supplementary Figure 1B). Among the other sncRNAs identified there were tRNAs, mitochondrial RNAs, and snoRNAs particularly in plasma exosomes. Consistently, considering those sncRNAs specific of each specimen, the highest number of sncRNAs was identified in urine (*n* = 127) and the same were grouped substantially apart from the other datasets in a PCA analysis using the sncRNA expression levels (Figure 3B).

PiR-31068 was the most abundant molecule in urine samples (Supplementary Figure 2D). The tRNA chr1.tRNA2-GlyCCC showed the highest expression levels among the sncRNAs specific in urine samples (median reads = 419 reads) while piR-43137 was the most abundant plasma exosome-specific sncRNA (median reads = 366 reads), and piR-36705 the most abundant stool-specific sncRNA (median reads = 131 reads) (Figure 3C and Supplementary Figure 2F). No specific sncRNAs of cervical scrapes were identified.

The specificity of these sets of sncRNAs was confirmed using a Random Forest classification algorithm which exactly classified 236 samples out of 243 (97.1%) (Supplementary Table 3B). The attribute selection analysis evidenced tRNAs chr19.tRNA2-GlyTCC, chr2.tRNA12-PseudoCTC, and chr6.tRNA150-MetCAT as the sncRNAs with the highest *merit* in the classification (Supplementary Table 3C).

Regression analysis between paired plasma exosome and stool samples or plasma exosome and urine samples from the same individuals showed a low coherent expression for sncRNAs detected (Supplementary Table 3D, 3E).

### Assessing inter-individual variability in sncRNA expression in each specimen type

**Figure 4:**
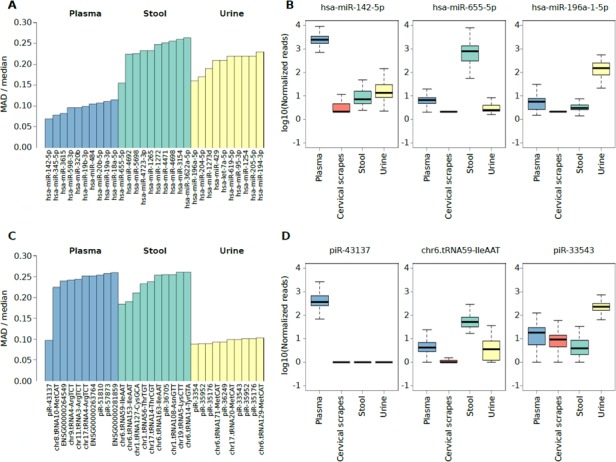
(**A**) Bar plot showing the top 10 miRNAs characterized by the lower ratio between the MAD and the median expression levels in plasma exosome, stool, or urine samples. (**B**) Box plot showing the log10 number of normalized reads supporting miRNAs characterized by the lower ratio between the MAD and the median expression level in plasma exosomes stool, or urine samples. (**C**) Bar plot showing the top 10 non-miRNA sncRNA species characterized by the lower ratio between the MAD and the median expression levels in plasma exosome, stool, or urine samples. (**D**) Box plot showing the log10 number of normalized reads supporting non-miRNA sncRNA species characterized by the lower ratio between the MAD and the median expression level in plasma exosomes stool, or urine samples.

Independently of the extensive intrinsic variability among subject’s extracellular RNA levels for each specimen, we selected the highly abundant sncRNAs with the lowest variable expression levels (i.e. potential reference sncRNAs) across all subjects. To achieve this, the highly-expressed miRNAs and sncRNAs specifically detected in plasma exosomes, stool, or urine (Figure 2A and 3A) characterized by the smallest expression variation in each specimen were identified by computing the median and the Median Absolute Deviation (MAD) of the expression levels (Supplementary Tables 2I and 3F). Specifically, the analysis highlighted miR-142-5p, miR-655-5p, and miR-196a-1-5p as potential *reference* miRNAs in plasma exosomes, stool, and urine, respectively (Figure 4A, 4B). Considering the isomiRs predicted for the reference miRNAs reported in Figure 4A, all the isomiRs predicted for reference miRNAs in plasma and urine were also identified in these sample types while no isomiRs were predicted for reference miRNAs in stool samples (Supplementary Table 2D). The analysis of *reference* non-miRNA sncRNAs highlighted piR-43137, chr6.tRNA59-IleAAT, and piR-33543 as the candidate sncRNAs for plasma exosome, stool, and urine samples, respectively (Figure 4C, 4D).

To further investigate the *reference* sncRNAs identified, an integrative analysis of public resources was performed (Supplementary Table 4A, 4B). Considering the top 10 reference miRNAs and sncRNAs characterized by the low ratio between MAD and median expression (Figure 4A, 4C), their expression was compared with RNA-Seq data from specimens collected in five independent studies and two databases publicly available. All the 10 most stably expressed miRNAs in plasma exosomes were also detected (average reads >20) in exosome data (analysed individuals, *n* = 40) from [15], plasma samples (*n* = 55) from [6], and venous blood samples data (*n* = 3) from [22]. Six out of 10 stably expressed urine miRNAs were detected in urine RNA (*n* = 4 and *n* = 55 analysed by [22] and [6], respectively). Interestingly, six of the 10 top miRNAs were also detected in samples from kidney (*n* = 11) or bladder (*n* = 2) small RNA-Seq data from DASHR database. The expression of the top 10 miRNAs in stool samples was not confirmed in stool data (*n* = 2) from [22], but two miRNAs were detected in colon samples (*n* = 8) by [23].

Among the reference non-miRNA sncRNAs, piR-62011 was detected as abundant in our plasma exosome data as well as plasma, serum and whole blood data from DASHR. Chr6.tRNA152-ValCAC was detected in our urine set and in small RNA-Seq data from DASHR kidney tissues [21]. All the others reference non-miRNA sncRNAs were generally associated with low expression in most of the datasets analysed.

## DISCUSSION

The study of the expression patterns of different sncRNAs in a wide spectrum of tissues, along with investigations into the functions of these molecules, is yielding novel insights in the fast-growing field of non-coding RNAs in the normal cell biology and pathogenesis. miRNAs have been extensively studied in the extracellular space but little is still known about the presence of other sncRNAs [15]. As diagnostic and therapeutic procedures move from biopsies in the direction to less invasive methodologies, sncRNAs analysed in different biospecimens represent attractive candidates as biomarkers for complex diseases [12].

In the present study, we investigated expression patterns of sncRNAs in different human biospecimens that could be easily and minimally invasively collected also in the context of screening programs. The data presented hereby were obtained from healthy subjects representing, on average, the steady state in normal conditions of the human organism.

The first analysis was focused on miRNA expression distribution across different investigated specimens. Globally, setting up an arbitrary threshold of median 20 reads, almost 400 miRNAs (out of an average of 1,046 unique miRNAs identified across specimens with at least one read) were detected, with many of them specific to one or few specimen types. A large set of miRNAs was expressed only in plasma exosomes (*n* = 155) while less miRNAs were private of stool or urine and only one of cervical scrapes. Plasma exosomes also shared several miRNAs with other specimens (particularly urine with 109 expressed miRNAs in common). Interestingly, considering the whole set of highly expressed miRNAs, it was possible to accurately group samples of the same biological type independently from the others. This aspect is important in search of specific biomarkers representing an altered status of a tissue in relation to a disease [24]. Conversely, eleven miRNAs presented a similar pattern of expression among all specimens. The most commonly investigated resulted miR-320a whose downregulation is associated with different diseases including cancer [25–30]. The relevance of an ubiquitous high expression of this miRNA related to a healthy status is supported by our findings as well. miR-589-5p, miR-636, and miR-4792 have been also described previously in other studies. miR-589-5p resulted a good inhibitor of MAP3K8 and suppressor of CD90+ cancer stem cells in hepatocellular carcinoma [31]. On the other hand, miR-636 was proposed as a good biomarker for several diseases in a large set of tissues and biofluids such as diabetic kidney disease [32], colorectal cancer [33], and pancreatic cancer [34]. Finally, miR-4792 was found dysregulated in oral submucous fibrosis [35], in nasopharyngeal carcinoma tissues [36] and in uterine leiomyoma [37]. Surprisingly, the rest of the commonly expressed miRNAs were not studied in detail before. Besides being found dysregulated in many studies in relation to different diseases, those miRNAs commonly expressed across different types of samples could be taken into consideration as multi-specimen markers. We have compared our results to those of available datasets on same specimens or anatomically-related tissues [6, 15, 16, 21, 22, 38]. The total number of reads obtained and the proportion of the detected sncRNA species is comparable to other studies previously published with the exception of the study of Yeri and colleagues that included YRNAs [6, 15, 38]. For instance, the high expression of the above mentioned miR-320a and miR-589-5p were also observed in all other datasets.

Notably, in our study, we could compare the co-expression of sncRNAs in plasma exosomes/urine or plasma exosomes/stool collected from the same subjects. Again, in the search of specific markers related to disease, it is important to have an overview on the similarities/differences across different biotypes at an individual level. Apparently, except for very few miRNAs mostly detected in urine/plasma, we could not observe any significant relationship between the expression of same sncRNAs in different biospecimens. This aspect is very important, in the sense that a multi-specimen miRNA panel may be more relevant for accurately describing a disease status, providing different miRNA behaviours across tissues. Similar findings were reported by us in a study on miRNA expression levels in both stool and whole plasma of healthy subjects with different dietary habits. Despite similar associations were observed between miRNA and diet (vegans, vegetarian vs omnivorous) or lifestyle habits, miRNA expression levels were not related between the two different specimens [39].

Since isomiRs have emerged as widely expressed in normal and cancer tissues [40, 41], we further investigated whether they were also detectable in the analysed specimens. As reported in Supplementary Table 2C–2D, many isomiRs were predicted in our datasets particularly in plasma and urine samples. Interestingly, among the 11 miRNAs commonly expressed in all specimens, eight were associated to an isomiR predicted in only one or two types of them.

miRNA profiling by NGS in different specimens in relation to healthy status and pathological conditions is becoming more and more frequent, especially in whole plasma [15]. Less explored is the field of other non-miRNA sncRNAs, although RNA sequencing potentialities, new annotation tools available and an increasing number of studies demonstrating their role in the normal physiology of the organism are appearing [42]. These ‘new’ small RNAs may play an important role in RNA silencing, micro-guarding and cancer [43]. In our study, we have confirmed that small RNA-Seq provide a huge number of reads not mapping to the miRNome in all type of samples analysed, particularly in stool. However, there is still not a consensus on how to comprehensively analyse these RNA molecules. In the present study, we focused on RNA species with a size between 30 to 70 nucleotides, due to the characteristics of the libraries prep kit employed, specific for small RNA sequencing. Considering these criteria, we have obtained a potential list of thousands of RNAs (>30,000) which we have used to filter the remapped reads after their annotation (from DASHR and GENCODE databases). Despite several different sncRNAs identifiable with our thresholds (misc_RNA, Mt_tRNA, piRNA, rRNA, snoRNA, snRNA, sRNA, tRNA), we have mainly identified piRNAs and tRNAs. In urine, we observed the largest number of “private” sncRNAs other than miRNAs (*n* = 127). Cervical scrapes had the less abundant number of these species and none of them was private. In total, 112 sncRNAs resulted expressed in all the biospecimens. Again, plasma exosome and urine samples shared many molecules in common (*n* = 150). Interestingly, as for miRNAs, also for the other sncRNAs, several molecules were characteristics of a single specimen while others were in common. Each body fluid appears to have clear differences in extracellular RNA expression profiles. For example, there appears to be a high proportion of piRNAs in urine samples, when compared with other RNA biotypes. This is quite similar to what observed by Yeri *et al.* [6] which observed an overrepresentation in urine of piRNAs and tRNAs. piRNAs hold great promise as potential biomarkers, owing to their sncRNA features such as small size, stability in biofluids and archival materials, and the variety of detection methods. Moreover, considering there are 10–25 times more piRNA species (20,000–50,000) than miRNAs, the impact of their deregulation is likely at least as relevant. Additionally, piRNA expression patterns have been shown to be deregulated in a variety of cancer types [44–46]. Recently, the study of tRNAs and their role in the regulation of gene expression is revealing new interesting aspects in molecular biology. tRNA-derived small RNAs, named tRNA halves (tiRNAs) and tRNA fragments (tRFs), have been reported to be abundant and their dysregulation to be associated with cancer [43]. Interestingly, we have not identified snoRNAs and other sncRNAs as reported in other studies [6, 47]. Better sncRNA tissue atlases that include more comprehensive profiles of the small RNA species will be necessary for better comparisons.

Expression patterns of miRNAs have been extensively studied but there is still controversy on the best endogenous control(s) to employ as reference in studies by RT-qPCR or microarray, especially when analysing biofluids [24]. An overview of the expression levels of sncRNAs in a large set of biofluids/biospecimen could provide a good base for the research of endogenous controls to be used in case-control studies when searching for sncRNAs as biomarkers of disease [24]. We propose miR-142-5p, miR-655-5p, and miR-196a-1-5p as miRNAs with a high and stable expression in plasma exosome, stool, and urine respectively, while piR-43137, chr6.tRNA59-IleAAT, and piR-33543 as the candidate references among other sncRNAs in plasma exosomes, stool, and urine respectively. miR-142-5p has been found dysregulated in plasma but not in exosomes [47–49] although it has been demonstrated that in rats the activation of the acute stress response modifies its profile in plasma exosomes [50]. miR-655-5p and miR-196a-1-5p have never been studied in stool and urine, except for miR-196a reported to be altered in focal segmental glomerulosclerosis [51]. Considering the top 10 *reference* miRNAs detected in plasma exosomes, stool, or urine sample group, we observed a general coherence between the specificity of isomiR and reference miRNA expression. The only exceptions were two 5′ variants of miR-204. However, these variants were detected by imposing two and three 5′ mismatches on a 14- and 15 nt sub-sequence of miR-204, respectively. The read alignment against such small sequences makes the read assignment less reliable reinforcing the hypothesis that a deeper sequencing depth is required to characterize properly the expression of these miRNA variants.

The biological samples used in the present work are very attractive for the research of non-invasive biomarkers. Blood plasma and urine belong to the group of easily accessible body fluids, and they are among the most frequently used diagnostic material for the development of surrogate cancer biomarkers [52, 53]. From the first work reporting the presence in plasma of miRNAs by Lawrie and colleagues [54], a growing number of studies have evaluated their expression in relation to a wide range of diseases and focused on the biology and features of circulating miRNAs [55]. Circulating miRNAs are considered as a tool employed in the horizontal gene transfer between cells within the tumor or between tumor and host cells: this is a strong biological rationale to use them as a new class of cancer biomarkers. miRNAs and other sncRNAs can be released by the cell by passive leakage into circulation. However, these molecular species can be released in a more active way from the cells by secretion of shedding microvesicles or exosomes containing free sncRNAs or in the form of ribonucleoprotein complexes [56]. Bladder cells are in direct contact with urine making this body fluid an ideal source for the detection of cancer biomarkers. Urine is collected noninvasively, and the procedure is relatively fast and cost-efficient compared with other clinical samples. In addition, sampling can be repeated at different times, and this makes urine an attractive candidate as a screening test for urogenital cancers that needs constant monitoring [53]. Stool has been extensively used as a potential substrate for developing non-invasive molecular screening tests for gastrointestinal diseases including colorectal cancer and for microbiome analyses. There is a rationale for determination of noncoding RNAs expression levels in stool which includes the observations that colonocytes are continuously shed into the faecal stream, with a periodicity of exfoliation roughly every 3–4 days. In addition, sncRNAs are extremely stable, enabling accurate and reproducible detection in the stool without need of special stabilization or logistical requirements. Conventional stool-based screening tests present several limitations including low sensitivity and specificity for advanced adenoma and pre-cancerous lesions. No optimal method has been established yet based on faecal DNA- and mRNA-based testing [57]. The role of diet and other lifestyle factors on miRNA and other sncRNA expression profiles in relation to disease risk is still scarcely explored [58]. Dietary components have been implicated in many pathways involved in diseases, including apoptosis, cell-cycle control, inflammation, and angiogenesis. Those pathways are also regulated by different RNAs [59]. Interestingly, recent discoveries point to a role of faecal miRNAs also introduced by the diet on shaping the human microbiota [60]. Cervical exfoliated cells are widely used in cervical cancer screening, both for HPV testing and Pap test. Recently, their use has been extended to miRNA analyses [61]. These few studies show that the potential application of miRNA detection in cervical exfoliated cells deserves further exploration, also as an additional option for triage of HPV-positive women in population-based screening.

We acknowledge that the present study has some limitations but also several strengths. Among the latter, we can consider the large number of samples sequenced, especially for plasma-exosomes, and the possibility to analyse different biospecimens of the same subjects to understand different/similar patterns according to tissue of origin. To our knowledge, we report the largest description of sncRNA data from plasma-derived exosome, as well as the first investigation of this kind on cervical scrape and stool samples by NGS in healthy subjects. Importantly, the outcomes of our study derive from samples analysed with the same protocols by the same operators and analysed by the same pipeline from raw sequencing data to final results. Other studies usually combine different datasets from different studies.

Among the limitations of our study, we can list that the library preparation is optimized for miRNAs while we have also adapted it for detecting a group of other sncRNAs. Additionally, we could not control analyses considering known potential confounders (age, gender) since not all the samples were provided with this information. Finally, some of the samples were investigated only in subjects of one gender only (i.e., urine in males only).

Small RNA-Seq holds promise for exhaustively analyse miRNAs and other sncRNAs in many different types of specimens, as we demonstrated in our study. These RNA molecules are currently investigated for their potential use as diagnostic/prognostic tools. The high resistance to degradation of sncRNAs makes these molecules particularly attractive for researchers that constantly cope with a wide range of incubations and storage conditions, as well as different origins of samples [62]. However, an optimization and standardization of both the biological and computational procedures to investigate sncRNA expression levels are necessary. Combining molecular aspects with bioinformatics and an epidemiological approach should provide stronger markers to be investigated specifically in particular biospecimens.

## MATERIALS AND METHODS

### Study participants

All samples included in the study were collected from healthy donors participating to different studies running in our laboratories who donated their blood (for plasma extraction), stool, and /or urine for research purposes [63, 64]. For cervical scrapes, samples were collected in the context of a national screening programme (New Technologies for Cervical Cancer screening (NTCC) study, [65]). All subjects provided written informed consent according to the Helsinki declaration. The design of the study was approved by the local Ethics Committees.

### Stool samples (study 1)

In a hospital-based study for colorectal cancer diagnosis, subjects resulting negative to colonoscopy and to any inflammatory disease were included in the present study. For the same individuals, we have collected also plasma samples (*n* = 39). Naturally evacuated stool samples were collected in special tubes with RNA stabilizing solution, returned at the time of performing colonoscopy and stored at –80°C until RNA extraction.

### Urine (study 2)

The study population included men recruited between the years 2008–2012 in the Turin Bladder Cancer Study (TBCS) who donated an aliquot of blood and urine. A full description of controls is available in Pardini *et al.* [66]. For almost all subjects, we have collected also plasma samples (*n* = 46).

Urine samples from each participant were collected in the morning, stored at 4°C until the processing consisting of centrifugation at 3,000g for 10 min. The urine supernatant aliquots were then transferred in tubes and stored at –80°C until use.

### Exosome isolation from plasma

In addition to the subjects described above for whom plasma samples were available (Study 1 and Study 2), we have included also 40 plasma samples collected from healthy blood donors for a Leukaemia study (Study 3).

For all subjects, human plasma samples were obtained from 5–8 ml of blood centrifuged for 10 min at 1000 rpm. Plasma aliquots (about 200–300 μl each) were then stored at –80°C until use. Exosomes were isolated from 200 μl of plasma using the ExoQuick exosome precipitation solution (System Biosciences, Mountain View, CA, USA) according to the manufacturer’s instructions with minor modifications. Briefly, the plasma was mixed with 50.4 μl of ExoQuick solution and refrigerated at 4°C overnight (at least 12 h). The mixture was then further centrifuged at 1500 g for 30 min. The exosome pellet was dissolved in 200 μl of nuclease free water; RNA was extracted immediately from the solution.

### Cervical scrapes

The study is nested in a large Italian multi-centre randomised controlled trial recruiting women in population-based screening programs that actively invite women aged 25–64 years (NTCC Study, [65]). NTCC recruitment was conducted between 2002 and 2004. In the present study, only samples from HPV negative women were included. Cervical scrape samples have been collected and stored in Specimen Transport Medium (STM), or RNA-later at –80°C until RNA extraction.

### RNA extraction and quality control

Total RNA from plasma exosomes was extracted with the miRNeasy plasma/serum mini kit (Qiagen) using the QiaCube extractor (Qiagen). RNA from stool was extracted using the Stool Total RNA Purification Kit (Norgen Biotek Corp). Total RNA from urine was extracted with Urine microRNA Purification kit (Norgen biotek corp), following the manufacturer’s standard protocol.

RNA from cervical scrape was extracted from samples stored in STM or RNA-later, using the miRCURY™ RNA Isolation Kit - Cell & Plant (Exiqon) following manufacturer`s protocol.

RNA quality and quantity was verified according to MIQE guidelines (http://miqe.gene-quantification.info/). For all samples, RNA concentration was quantified by Qubit^®^ 2.0 Fluorometer with Qubit^®^ microRNA Assay Kit (Invitrogen).

### Library preparation for small RNA-Seq

Small RNA transcripts were converted into barcoded cDNA libraries. Library preparation was performed with the NEBNext Multiplex Small RNA Library Prep Set for Illumina (New England BioLabs Inc., USA). For each library, 6 μL of RNA (min 35 ng) were used in all the experimental procedures as starting material. Each library was prepared with a unique indexed primer so that the libraries could all be pooled into one sequencing lane. Multiplex adaptor ligations, reverse transcription primer hybridization, reverse transcription reaction and PCR amplification were performed according to the protocol for library preparation (Protocol E7330, New England BioLabs Inc., USA). After PCR amplification, the cDNA constructs were purified with the QIAQuick PCR Purification Kit (Qiagen, Germany) following the modifications suggested by the NEBNext Multiplex Small RNA Library Prep Protocol and loaded on the Bioanalyzer 2100 (Agilent, Germany) using the DNA High Sensitivity Kit (Agilent, Germany) according to the manufacturer’s protocol. Libraries were pooled together (24plex) and further purified with a gel size selection.

A concluding Bioanalyzer 2100 run with the High Sensitivity DNA Kit (Agilent Technologies, Germany) that allows the analysis of DNA libraries regarding size, purity and concentration completed the workflow of library preparation. The obtained sequence libraries were subjected to the Illumina sequencing pipeline, passing through clonal cluster generation on a single-read flow cell (Illumina Inc., USA) by bridge amplification on the cBot (TruSeq SR Cluster Kit v3-cBOT-HS, Illumina Inc., USA) and 50 cycles sequencing-by-synthesis on the HiSeq 2000 (Illumina Inc., USA) (in collaboration with EMBL, Heidelberg, Germany).

### Computational analyses (additional information in Supplementary Material)

#### Analysis of miRNAs

miRNA data analysis was performed following the optimized workflow proposed in [19]. The obtained FASTQ files from small RNA-seq were quality-checked using FastQC software.

Reads shorter than 14 nucleotides were discarded from the analysis; the remaining reads were clipped from the adapter sequences using Cutadapt software (http://journal.embnet.org/index.php/embnetjournal/article/view/200). The trimmed reads were mapped against the precursor miRNA sequences downloaded from miRBase (Release 21) by the Shrimp algorithm. A matrix of integer values called counting matrix was created.

Since plasma datasets were generated in independent studies and presented a large variability, a SVA [67] was performed to correct the read counts. IsomiR analysis was performed using isomiRID algorithm [68] in default settings. A maximum of three mismatches between reads and reference miRNA sequences was considered for the analysis.

#### Analysis of other sncRNAs

The set of small RNA-Seq reads not aligned by SHRiMP over miRNA sequences were aligned against human genomic sequence hg38 (GRCh38) using Bowtie2 v2.2.7 in default settings [69]. Reads alignment files were used to quantify the expression of ncRNA annotations from Gencode v24 [70] and DASHR database [21]. The annotations with median reads greater than 20 were selected. Then, read counts were normalized by computing the library size factor [71]. The SVA [67] was performed to correct the read counts of plasma studies.

#### Bioinformatic tools and data integration

The list and the expression levels of sncRNAs identified in the different specimen types were compared using Venn diagrams and *heatmap.2* R functions. PCA analysis was performed using *prcomp* R function and *autoplot* function from *ggfortify* R package. The contribution of each sncRNA expression level to the classification of specimen type was evaluated using Weka 3.6.12 [72]. miRNA functional enrichment analysis was performed using EnrichR web tool [73] on the list of validated miRNA targets annotated in miRWalk 2.0 database [74].

The set of sncRNAs identified in this study was compared with public lists sncRNAs detected in specimens and tissues from healthy individuals as reported in supplementary materials of target publications and databases.

## SUPPLEMENTARY MATERIALS FIGURES AND TABLES





































## Abbreviations

isomiR: isoform of miRNAs
miRNA: microRNA
piRNAs: Piwi-interacting RNA
tRNAs: transfer RNA
RT-qPCR: Quantitative reverse transcription PCR
sncRNA: small non-coding RNA
snoRNAs: small nucleolar RNAs
NGS: Next-Generation Sequencing
RNA-Seq: RNA-Sequencing
HPV: Human Papilloma Virus
DASHR: Database of Small Human non-coding RNAs
PCA: principal component analysis
mRNA: messenger RNA
MAD: Median Absolute Deviation
tiRNAs: tRNA halves
tRFs: tRNA fragments
TBCS: Turin Bladder Cancer Study
NTCC: New Technologies for CC screening
STM: Specimen Transport Medium
GRCh38: Genome Reference Consortium Human Build 38
SVA: Surrogate Variable Analysis
