# Supplementary material for: Small non-coding RNA profiling in human biofluids and surrogate tissues from healthy individuals: description of the diverse and most represented species
Source: Oncotarget. 2017 Dec 14;9(3):3097–111. doi: 10.18632/oncotarget.23203 (PMC5790449; doi:10.18632/oncotarget.23203)
Supplement: Supplementary file 1 [file oncotarget-09-3097-s001.pdf]

# Small non-coding RNA profiling in human biofluids and surrogate tissues from healthy individuals: description of the diverse and most represented species

## SUPPLEMENTARY MATERIALS

### Analysis of miRNAs

miRNA data analysis was performed following the optimized workflow proposed in [1]. The obtained FASTQ files were quality-checked using FastQC software (http://www.bioinformatics.babraham.ac.uk/projects/fastqc/). The base quality and the N content features were also considered: all files passed both these checks.

Reads shorter than 14 nucleotides were discarded from the analysis; the remaining reads were clipped from the adapter sequences using Cutadapt software (http://journal.embnet.org/index.php/embnetjournal/article/view/200). In Cutadapt, the maximum error rate in terms of mismatches, insertions and deletions was set to 0.15. The trimmed reads were mapped against the precursor miRNA sequences downloaded from miRBase (Release 21) using the Shrimp algorithm [2] setting the options for miRNA sequences alignment. The use of precursor miRNAs as reference guarantees a precise and specific count detection.

Only those reads with maximum 2 mismatches were retained. After reads filtering steps, a matrix of integer values called counting matrix was created. The value in the i-th row and the j-th column of the matrix reports how many reads have been unambiguously assigned to mature miRNA i in the sample j.

Since these datasets were generated in independent studies a Surrogate Variable Analysis (SVA) [3] was performed to correct the read counts. The analysis was performed using the *svaseq* function of the package and by setting the number of surrogate variable equal to three.

The read count tables from the three studies of plasma exosomes samples were merged into a single study.

### Detection of isomiRNAs

IsomiR analysis was performed using isomiRID algorithm [4] using the default settings. Only isomiRs associated with a median number of reads greater than 20 in at least one biospecimen were considered. A maximum of three mismatches between reads and reference miRNA sequences was considered for the analysis.

### Analysis of other sncRNAs

The set of small RNA-Seq reads not aligned by SHRiMP to miRNA sequences were aligned against human genomic sequence hg38 (GRCh38) using Bowtie2 v2.2.7 in default settings [5].

Reads alignment files were used to quantify the expression of ncRNA annotations from Gencode v24 [6] and Database of small human non-coding RNAs (DASHR) database [7]. Specifically, Gencode v24 database was used to isolate the ncRNA annotations shorter than 70 bp. According with this threshold, 276 ncRNAs were isolated. DASHR was used to identify the set of piRNA (average length 31+/−1 bp) and tRNA (average length 74+/−7 bp) annotations. In total, 34,175 piRNA and 643 tRNA annotations were isolated from DASHR (Supplementary Table 1C).

Reads mapping to ncRNA loci were counted using *featureCounts* algorithm from Subreads v1.5.0 package (Liao *et al*., 2014). The algorithm was applied with options *–O* and *–M* and counting separately reads mapped on Gencode v24, piRNA and tRNA genes.

To identify the ncRNAs expressed in each biospecimen, the annotations with median reads greater than 20 were selected. Then, read counts were normalized by computing the library size factor [8]. The read count tables from the three studies of plasma exosomes samples were merged into a single study. Since these datasets were generated in independent studies a SVA [3] was performed to correct the read counts. The analysis was performed using the *svaseq* function of the package and by setting the number of surrogate variable equal to three.

### External data integration

The set of sncRNAs identified in this study was compared with public lists of sncRNAs detected in specimens and tissues from healthy individuals as reported in supplementary materials of target publications and databases. Specifically, normalized expression from DASHR databases were used to compare the expression of miRNAs and other sncRNAs in plasma, serum, and eight human tissues related to the biospecimens analysed in this study. Supplementary data from [9] were used to verify miRNA expression levels in plasma and urine samples. Data from [10] and [11] were used to assess the expression of miRNAs in normal colon tissues. The expression levels of miRNAs and tRNAs in 40 plasma samples were retrieved from [12]. miRNA expression levels from different specimens were retrieved from GSE85830 [13].

ExoCarta database [14] annotations were used to retrieve information about miRNA expression in extracellular vesicles.

### Bioinformatic tools

The list and the expression levels of sncRNAs identified in the different specimen types were compared using Venn diagrams and *heatmap.2* R functions. PCA analysis was performed using *prcomp* R function and *autoplot* function from *ggfortify* R package. The contribution of each sncRNAs expression level to the classification of specimen type was evaluated using Weka 3.6.12 [15]. The Weka *RandomForest* classifier was applied in default settings and 10-fold cross-validation. The contribution of each covariate to the classification results was evaluated using Weka *ChiSquareAttributeEval*. This methodology is based on the independence of the occurrence of a specific attribute (sncRNA expression) and the occurrence of a specific class (specimen type). The miRNA functional enrichment analysis was performed using EnrichR web tool [16] on the list of validated miRNA targets annotated in miRWalk 2.0 database [17].

## REFERENCES


1. Cordero F, Beccuti M, Arigoni M, Donatelli S, Calogero RA. Optimizing a massive parallel sequencing workflow for quantitative miRNA expression analysis. PLoS One. 2012; 7:e31630. https://doi.org/10.1371/journal.pone.0031630.
2. Rumble SM, Lacroute P, Dalca AV, Fiume M, Sidow A, Brudno M. SHRiMP: accurate mapping of short color-space reads. PLOS Comput Biol. 2009; 5:e1000386. https://doi.org/10.1371/journal.pcbi.1000386.
3. Leek JT, Johnson WE, Parker HS, Jaffe AE, Storey JD. The sva package for removing batch effects and other unwanted variation in high-throughput experiments. Bioinformatics. 2012; 28:882–83. https://doi.org/10.1093/bioinformatics/bts034.
4. de Oliveira LF, Christoff AP, Margis R. isomiRID: a framework to identify microRNA isoforms. Bioinformatics. 2013; 29:2521–23. https://doi.org/10.1093/bioinformatics/btt424.
5. Langmead B, Salzberg SL. Fast gapped-read alignment with Bowtie 2. Nat Methods. 2012; 9:357–59. https://doi.org/10.1038/nmeth.1923.
6. Harrow J, Frankish A, Gonzalez JM, Tapanari E, Diekhans M, Kokocinski F, Aken BL, Barrell D, Zadissa A, Searle S, Barnes I, Bignell A, Boychenko V, et al. GENCODE: the reference human genome annotation for The ENCODE Project. Genome Res. 2012; 22:1760–74. https://doi.org/10.1101/gr.135350.111.
7. Leung YY, Kuksa PP, Amlie-Wolf A, Valladares O, Ungar LH, Kannan S, Gregory BD, Wang LS. DASHR: database of small human noncoding RNAs. Nucleic Acids Res. 2016; 44:D216–22. https://doi.org/10.1093/nar/gkv1188.
8. Love MI, Huber W, Anders S. Moderated estimation of fold change and dispersion for RNA-seq data with DESeq2. Genome Biol. 2014; 15:550. https://doi.org/10.1186/s13059-014-0550-8.
9. Yeri A, Courtright A, Reiman R, Carlson E, Beecroft T, Janss A, Siniard A, Richholt R, Balak C, Rozowsky J, Kitchen R, Hutchins E, Winarta J, et al. Total Extracellular Small RNA Profiles from Plasma, Saliva, and Urine of Healthy Subjects. Sci Rep. 2017; 7:44061. https://doi.org/10.1038/srep44061.
10. Neerincx M, Sie DL, van de Wiel MA, van Grieken NC, Burggraaf JD, Dekker H, Eijk PP, Ylstra B, Verhoef C, Meijer GA, Buffart TE, Verheul HM. MiR expression profiles of paired primary colorectal cancer and metastases by next-generation sequencing. Oncogenesis. 2015; 4:e170. https://doi.org/10.1038/oncsis.2015.29.
11. Hamfjord J, Stangeland AM, Hughes T, Skrede ML, Tveit KM, Ikdahl T, Kure EH. Differential expression of miRNAs in colorectal cancer: comparison of paired tumor tissue and adjacent normal mucosa using high-throughput sequencing. PLoS One. 2012; 7:e34150. https://doi.org/10.1371/journal.pone.0034150.
12. Freedman JE, Gerstein M, Mick E, Rozowsky J, Levy D, Kitchen R, Das S, Shah R, Danielson K, Beaulieu L, Navarro FC, Wang Y, Galeev TR, et al. Diverse human extracellular RNAs are widely detected in human plasma. Nat Commun. 2016; 7:11106. https://doi.org/10.1038/ncomms11106.
13. Seashols-Williams S, Lewis C, Calloway C, Peace N, Harrison A, Hayes-Nash C, Fleming S, Wu Q, Zehner ZE. High-throughput miRNA sequencing and identification of biomarkers for forensically relevant biological fluids. Electrophoresis. 2016; 37:2780–88. https://doi.org/10.1002/elps.201600258.
14. Keerthikumar S, Chisanga D, Ariyaratne D, Al Saffar H, Anand S, Zhao K, Samuel M, Pathan M, Jois M, Chilamkurti N, Gangoda L, Mathivanan S. ExoCarta: A Web-Based Compendium of Exosomal Cargo. J Mol Biol. 2016; 428:688–92. https://doi.org/10.1016/j.jmb.2015.09.019.
15. Frank E, Hall M, Trigg L, Holmes G, Witten IH. Data mining in bioinformatics using Weka. Bioinformatics. 2004; 20:2479–81. https://doi.org/10.1093/bioinformatics/bth261.
16. Kuleshov MV, Jones MR, Rouillard AD, Fernandez NF, Duan Q, Wang Z, Koplev S, Jenkins SL, Jagodnik KM, Lachmann A, McDermott MG, Monteiro CD, Gundersen GW, Ma'ayan A. Enrichr: a comprehensive gene set enrichment analysis web server 2016 update. Nucleic Acids Res. 2016; 44:W90-7. https://doi.org/10.1093/nar/gkw377.
17. Dweep H, Gretz N. miRWalk2.0: a comprehensive atlas of microRNA-target interactions. Nat Methods. 2015; 12:697. https://doi.org/10.1038/nmeth.3485.

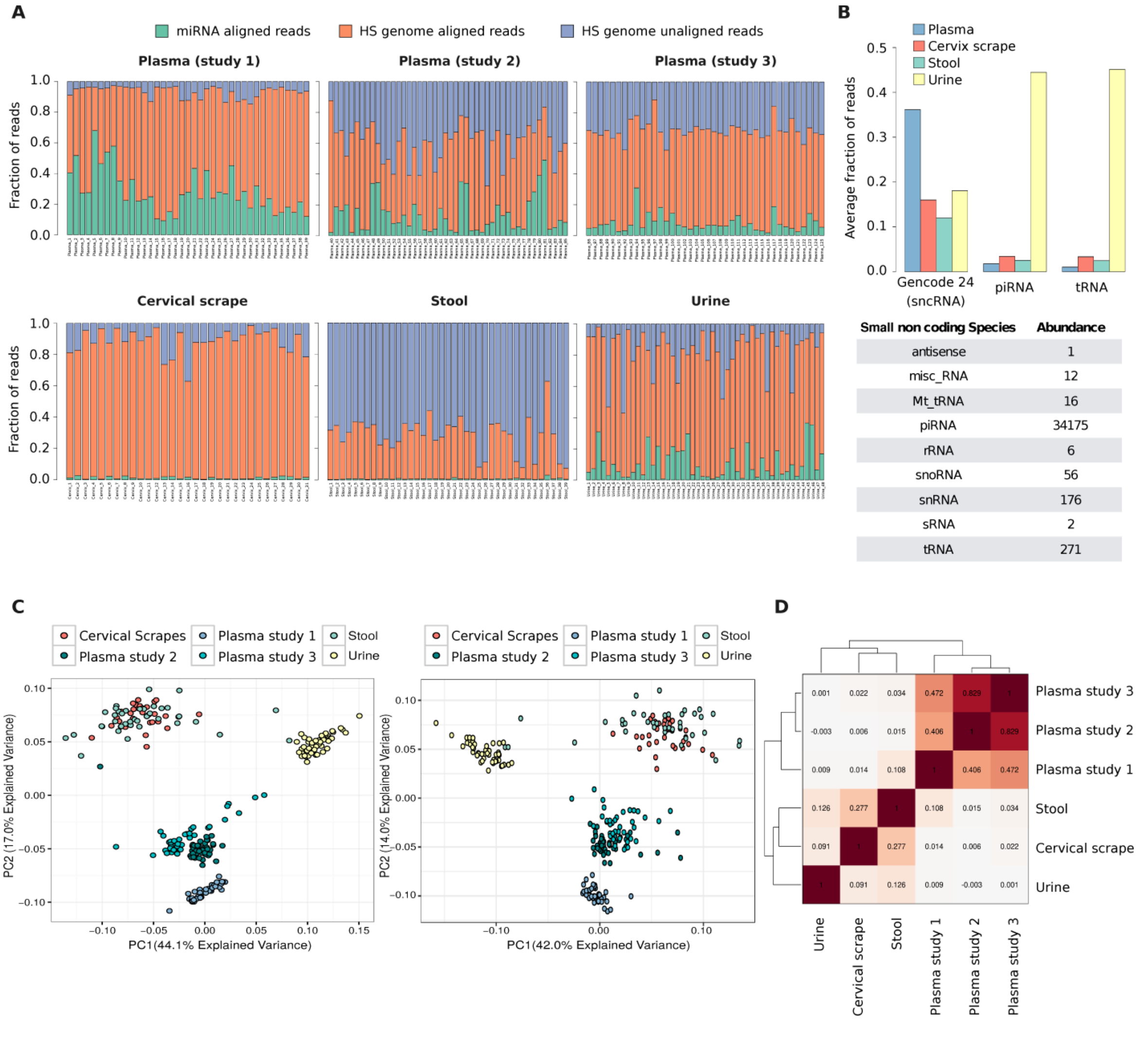


| Small non coding Species | Abundance |
|---|---|
| antisense | 1 |
| misc_RNA | 12 |
| Mt_tRNA | 16 |
| piRNA | 34175 |
| rRNA | 6 |
| snoRNA | 56 |
| snRNA | 176 |
| sRNA | 2 |
| tRNA | 271 |

**Supplementary Figure 1:** (**A**) Stacked bar plot reporting the fraction of small RNA-Seq reads aligned on miRNA annotations (green), unmapped on miRNA annotations but mapped on human genome (red), and reads unmapped on both miRNA annotations and the human genome (blue). (**B**) Bar plot showing the fraction of reads assigned to sncRNAs (from Gencode v24), piRNA, or tRNA annotations in each group of biospecimens. The abundance of sncRNAs is reported in the table below. (**C**) PCA plot before (left) and after SVA correction (right) showing with different colours the RNA-Seq datasets from different specimen type. (**D**) Heat map showing the average expression correlation computed across datasets from each type of specimen analysed. HS = Homo sapiens.

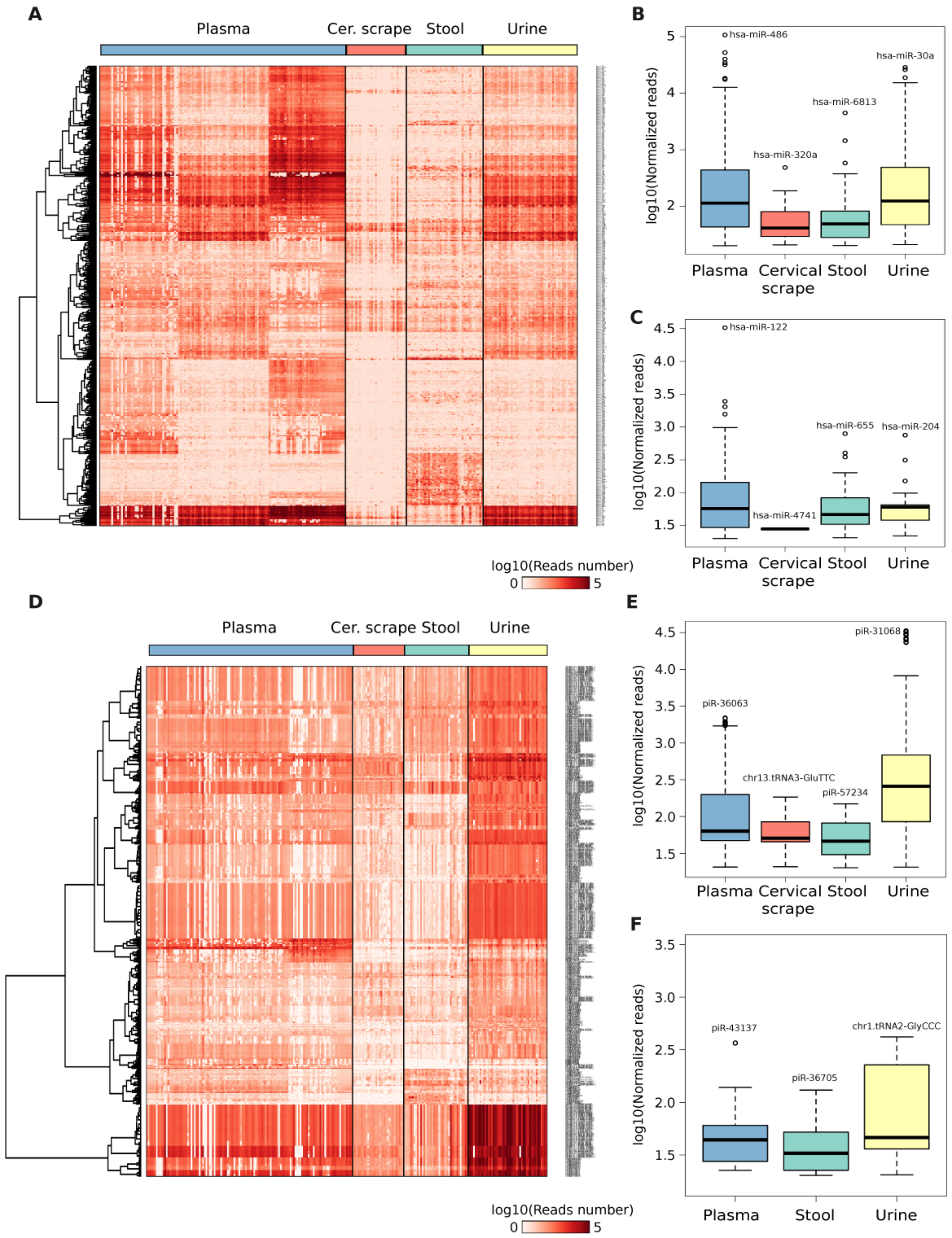


**Supplementary Figure 2:** (**A**) Heat map showing the log10 number of normalized reads supporting all the miRNAs identified in analysed samples. (**B–C**) Box plot reporting the median normalized number or reads of all (B) or biospecimen-specific (C) miRNAs detected. (**D**) Heat map showing the log10 number of normalized reads supporting all the non-miRNA sncRNAs species identified in analysed samples. (**E–F**) Box plot reporting the median normalized number or reads of all (E) or specimen-specific (F) non-miRNA sncRNA species detected.

**Supplementary Table 1A: Summary of datasets before and after alignment.** See Supplementary_Table_1A

**Supplementary Table 1B: Summary of all quality control data for each sample in each dataset.** See Supplementary_Table_1B

**Supplementary Table 1C: All the annotated species detected in the study as described in GENCODE and DASHR database.** See Supplementary_Table_1C

**Supplementary Table 2A: List of all miRNAs quantified in at least one specimen.** See Supplementary_Table_2A

**Supplementary Table 2B: Enrichment analyses of genes targeted by miRNAs commonly expressed in all specimens.** See Supplementary_Table_2B

**Supplementary Table 2C: Weka analysis: random forest classifier results to identify discriminative miRNAs able to distinguish the studied specimens**

| **Summary** | |
|---|---|
| Correctly Classified Instances | 242 (99.59%) |
| Incorrectly Classified Instances | 1 (0.41%) |
| Kappa statistic | 0.9937 |
| Mean absolute error | 0.0389 |
| Root mean squared error | 0.0793 |
| Relative absolute error | 11.87% |
| Root relative squared error | 19.61% |
| Total Number of Instances | 243 |

**Detailed Accuracy By Class**

| TP Rate | FP Rate | Precision | Recall | F-Measure | ROC area | Class |
|---|---|---|---|---|---|---|
| 1 | 0 | 1 | 1 | 1 | 1 | Plasma |
| 1 | 0 | 1 | 1 | 1 | 1 | Cervical scrapes |
| 0.974 | 0 | 1 | 0.974 | 0.987 | 1 | Stool |
| 1 | 0.005 | 0.98 | 1 | 0.99 | 1 | Urine |

**Confusion Matrix**

| a | b | c | d | <-- classified as |
|---|---|---|---|---|
| 125 | 0 | 0 | 0 | a = Plasma |
| 0 | 31 | 0 | 0 | b = Cervix |
| 0 | 0 | 38 | 1 | c = Stool |
| 0 | 0 | 0 | 48 | d = Urine |

**Supplementary Table 2D: List of all detected miRNAs classified in accordance to their discriminative capacity.** See Supplementary_Table_2D

**Supplementary Table 2E: Correlation analyses in plasma and stool of miRNAs expressed in multiple specimens**

| miRNA | Specimens | Plasma-Stool correlation | *p*-value |
|---|---|---|---|
| hsa-miR-7-1 | Plasma exosomes, Stool, Urine | 0.4014 | 0.0125 |
| hsa-miR-181d | Plasma exosomes, Stool | 0.287 | 0.0806 |
| hsa-miR-192 | Plasma exosomes, Stool, Urine | 0.2717 | 0.0989 |
| hsa-miR-92a-1 | Plasma exosomes, Stool, Urine | 0.2689 | 0.1026 |
| hsa-miR-7641-2 | Common | 0.133 | 0.4260 |
| hsa-miR-30b | Plasma exosomes, Stool, Urine | 0.1228 | 0.4628 |
| hsa-miR-4302 | Plasma exosomes, Stool | 0.1045 | 0.5325 |
| hsa-miR-4709 | Common | 0.0966 | 0.5641 |
| hsa-miR-7851 | Plasma exosomes, Stool, Urine | 0.0735 | 0.6609 |
| hsa-miR-3665 | Plasma exosomes, Stool, Urine | 0.0724 | 0.6656 |
| hsa-miR-320a | Common | 0.0633 | 0.7056 |
| hsa-miR-3125 | Plasma exosomes, Stool, Urine | 0.0624 | 0.7098 |
| hsa-miR-6087 | Plasma exosomes, Stool, Urine | 0.0584 | 0.7277 |
| hsa-miR-4419a | Common | 0.0444 | 0.7912 |
| hsa-miR-636 | Common | 0.0385 | 0.8186 |
| hsa-miR-182 | Plasma exosomes, Stool, Urine | 0.037 | 0.8252 |
| hsa-miR-7641-1 | Common | 0.0351 | 0.8343 |
| hsa-miR-3160-2 | Plasma exosomes, Stool | 0.0194 | 0.9077 |
| hsa-miR-4326 | Plasma exosomes, Stool | 0.0154 | 0.9270 |
| hsa-miR-4792 | Common | 0.0007 | 0.9967 |
| hsa-miR-378a | Plasma exosomes, Stool, Urine | –0.0253 | 0.8800 |
| hsa-miR-654 | Plasma exosomes, Stool | –0.0309 | 0.8538 |
| hsa-miR-1304 | Plasma exosomes, Stool, Urine | –0.0322 | 0.8476 |
| hsa-miR-203a | Plasma exosomes, Stool, Urine | –0.0383 | 0.8193 |
| hsa-miR-125b-1 | Plasma exosomes, Stool, Urine | –0.042 | 0.8023 |
| hsa-miR-1911 | Plasma exosomes, Stool, Urine | –0.049 | 0.7703 |
| hsa-miR-4448 | Plasma exosomes, Stool, Urine | –0.0494 | 0.7682 |
| hsa-miR-3960 | Common | –0.0545 | 0.7451 |
| hsa-miR-589 | Common | –0.0697 | 0.6776 |
| hsa-miR-6813 | Plasma exosomes, Stool, Urine | –0.0869 | 0.6041 |
| hsa-miR-1273a | Common | –0.0882 | 0.5986 |
| hsa-miR-588 | Plasma exosomes, Stool | –0.0903 | 0.5896 |
| hsa-miR-133a-2 | Plasma exosomes, Stool | –0.0904 | 0.5894 |
| hsa-miR-4284 | Plasma exosomes, Stool | –0.099 | 0.5543 |
| hsa-miR-222 | Plasma exosomes, Stool, Urine | –0.1144 | 0.4942 |
| hsa-miR-3168 | Plasma exosomes, Stool | –0.1271 | 0.4469 |
| hsa-miR-1246 | Plasma exosomes, Stool, Urine | –0.1331 | 0.4256 |
| hsa-miR-4497 | Common | –0.1426 | 0.3930 |
| hsa-miR-5094 | Plasma exosomes, Stool | –0.1531 | 0.3587 |
| hsa-miR-98 | Plasma exosomes, Stool, Urine | –0.1561 | 0.3492 |
| hsa-miR-1307 | Plasma exosomes, Stool, Urine | –0.161 | 0.3343 |
| hsa-miR-21 | Plasma exosomes, Stool, Urine | –0.1751 | 0.2930 |
| hsa-miR-143 | Plasma exosomes, Stool, Urine | –0.1875 | 0.2597 |
| hsa-miR-3976 | Plasma exosomes, Stool, Urine | –0.1988 | 0.2314 |
| hsa-miR-647 | Plasma exosomes, Stool | –0.204 | 0.2191 |
| hsa-miR-8072 | Plasma exosomes, Stool, Urine | –0.2771 | 0.0922 |
| hsa-miR-186 | Plasma exosomes, Stool, Urine | –0.2859 | 0.0819 |

**Supplementary Table 2F: Correlation analyses in plasma and urine of miRNAs expressed in multiple specimens.** See Supplementary_Table_2F

**Supplementary Table 2G: List of isomiRs predicted by isomiRID with associated median number of supporting reads.** See Supplementary_Table_2G

**Supplementary Table 2H: List of isomiRs predicted for common or reference miRNAs.** See Supplementary_Table_2F

**Supplementary Table 2I: Highly abundant miRNAs with the lowest variable expression for the estimation of inter-individual variability in each specimen type.** See Supplementary_Table_2I

**Supplementary Table 3A: List of sncRNAs other than miRNAs quantified in at least one specimen.** See Supplementary_Table_3A

**Supplementary Table 3B: Weka analysis: random forest classifier results to identify discriminative sncRNAs able to distinguish the studied specimens**

| **Summary** | |
|---|---|
| Correctly Classified Instances | 236 (97.12%) |
| Incorrectly Classified Instances | 7 (2.88%) |
| Kappa statistic | 0.96 |
| Mean absolute error | 0.05 |
| Root mean squared error | 0.11 |
| Relative absolute error | 16.34 |
| Root relative squared error | 27.88 |
| Total Number of Instances | 243 |

**Detailed Accuracy By Class**

| TP Rate | FP Rate | Precision | Recall | F-Measure | ROC area | Class |
|---|---|---|---|---|---|---|
| 0.976 | 0.008 | 0.992 | 0.976 | 0.984 | 1 | Plasma |
| 1 | 0.014 | 0.912 | 1 | 0.954 | 1 | Cervical scrapes |
| 0.923 | 0.005 | 0.973 | 0.923 | 0.947 | 1 | Stool |
| 0.979 | 0.01 | 0.959 | 0.979 | 0.969 | 0.999 | Urine |

**Confusion Matrix**

| a | b | c | d | <-- classified as |
|---|---|---|---|---|
| 122 | 0 | 1 | 2 | a = Plasma |
| 0 | 31 | 0 | 0 | b = Cervix |
| 0 | 3 | 36 | 0 | c = Stool |
| 1 | 0 | 0 | 47 | d = Urine |

**Supplementary Table 3C: List of all detected sncRNAs classified in accordance to their discriminative capacity.** See Supplementary_Table_3C

**Supplementary Table 3D: Correlation analyses in plasma and stool of sncRNAs expressed in multiple specimens.** See Supplementary_Table_3D

**Supplementary Table 3E: Correlation analyses in plasma and urine of sncRNAs expressed in multiple specimens.** See Supplementary_Table_3E

**Supplementary Table 3F: Highly abundant sncRNAs with the lowest variable expression for the estimation of inter-individual variability in each specimen type**. See Supplementary_Table_3F

**Supplementary Table 4A: A comparison of miRNAs detected in the study as reference and the data available on public databases.** See Supplementary_Table_4A

**Supplementary Table 4B: A comparison of non-miRNAs sncRNAs detected in the study as reference and the data available on public databases.** See Supplementary_Table_4B
